# Supplementary material for: Decarbonization, population disruption and resource inventories in the global energy transition
Source: Nat Commun. 2022 Dec 15;13:7674. doi: 10.1038/s41467-022-35391-2 (PMC9755273; doi:10.1038/s41467-022-35391-2)
Supplement: Supplementary file 1 — Supplementary Information [file 41467_2022_35391_MOESM1_ESM.pdf]

Supplementary Information

Decarbonization, population disruption and resource inventories in the global energy transition

Kamila Svobodova, John R. Owen, Deanna Kemp, Vítězslav Moudrý, Éléonore Lèbre, Martin Stringer, and Benjamin K. Sovacool

Supplementary Information 1

Mine-town system examples. The mine-town systems listed below present three examples, where the potential impacts of the transition are described, considering the socio-economic conditions of the populations in these mine-town systems. While Example 1 (EMalahleni, South Africa) and Example 3 (Mackay, Australia) show the mine-town systems with prevailing coal phase-out transition, communities in Example 2 (Salta, Argentina) may face a rapid ETM phase-in transition.

See next page.

1 | MINE-TOWN SYSTEM EXAMPLE

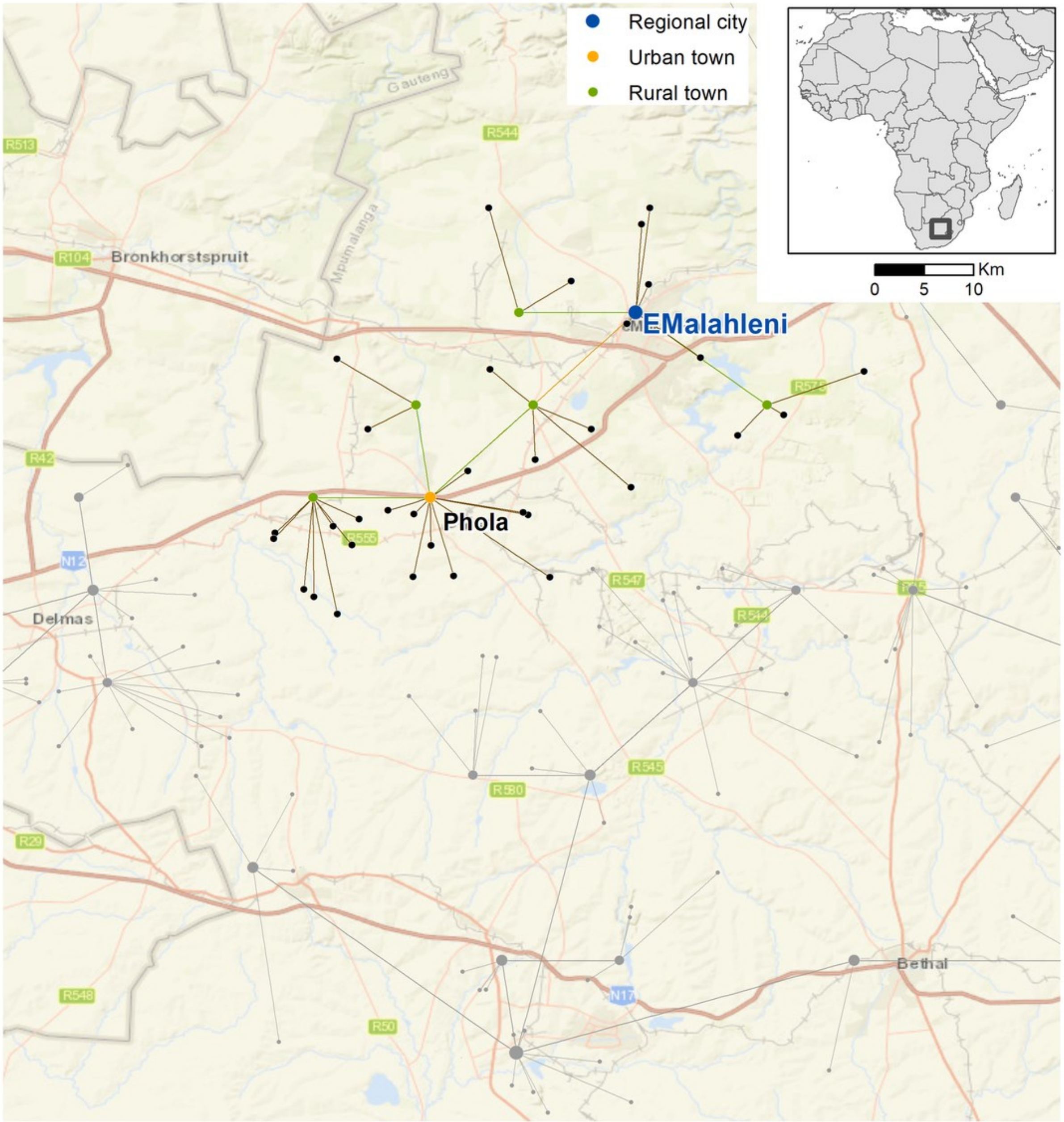

Town system:

- 1 regional city = EMalahleni
- 1 urban town = Phola
- 5 rural towns
- 57,500 min. estimated population

Resource Inventories:

- A total of 34 mining projects
- 34 coal mining projects in Resource Inventory I: pre-operating 8; operating 23; closed 3
- 0 ETM projects in Resource Inventories II and III
- 0 other commodities

EMALAHLENI, SOUTH AFRICA

EMalahleni mine-town system is located in the province of Mpumalanga, South Africa. The regional city EMalahleni (population 108,673; 2011 census[1]), previously known as Witbank, is administered as part of the larger Emalahleni Local Municipality (population 395,466; 2011 census). The EMalahleni coalfield is historically the most important coal-producing region in South Africa, with the largest concentration of power stations in the country (SACN 2014). Most of the coal extraction takes place through open-cast mining methods. Currently, much of the farmland in EMalahleni Municipality, that has not been mined, is held by the mining companies and leased to farmers[2]. The contribution of mining to the city's gross value added increased from 34% to 47% from 1996 to 2011, while employment in mining has fluctuated between 21% and 24% in the municipality. There is a 27,3% unemployment rate and 26.6% of households have no access to electricity for lighting[1]. Households in Mpumalanga Province

have the largest increase of 148.9%, in comparison to the other eight provinces (R77,609; 2011 census[3]). A similar trend is evident across the EMalahleni mine-town system.

MINE-TOWN SYSTEM EMALAHLENI

The EMalahleni mine-town system is built on coal Resource Inventory I. The regional city of EMalahleni has been sustained by coal mining and related industries and has developed into a strong regional city, offering the full spectrum of business and social activities for the six smaller settlements in the mine-town system. Coal phase-out may therefore directly impact the entire system with a minimal population of 57,500 people.

PHASE-IN, PHASE-OUT ASSUMPTIONS AND POTENTIAL IMPACTS

Currently, coal mining contributes 47% of EMalahleni's GVA and is estimated to continue in a similar way for the next decade[4]. Under the coal phase-out assumption, a decline in an industry that plays such a central role in the regional and national economies may impact not only the population of the mine-town system but also the province and the nation. The rapid economic development of EMalahleni had social and environmental consequences. There was rapid population growth, putting elementary services such as water, electricity and roads under strain. Environmental degradation, as a result of mining and related industries, has been a major threat locally and to the watershed[2]. Mine closures may exacerbate these already existing issues, causing long term social consequences. These may include increased unemployment, poverty, out-migration, closure of enterprises serving the mines, disintegration of social structures, decline of infrastructure and the cessation of essential services. The absence of the mine closure legislative framework due to capacity constraints of the South Africa government[5] may lead to the five rural towns in the EMalahleni mine-town system being abandoned, with cascading impacts on Phola and EMalahleni through corresponding in- and out-migration of their residents.

2 | MINE-TOWN SYSTEM EXAMPLE

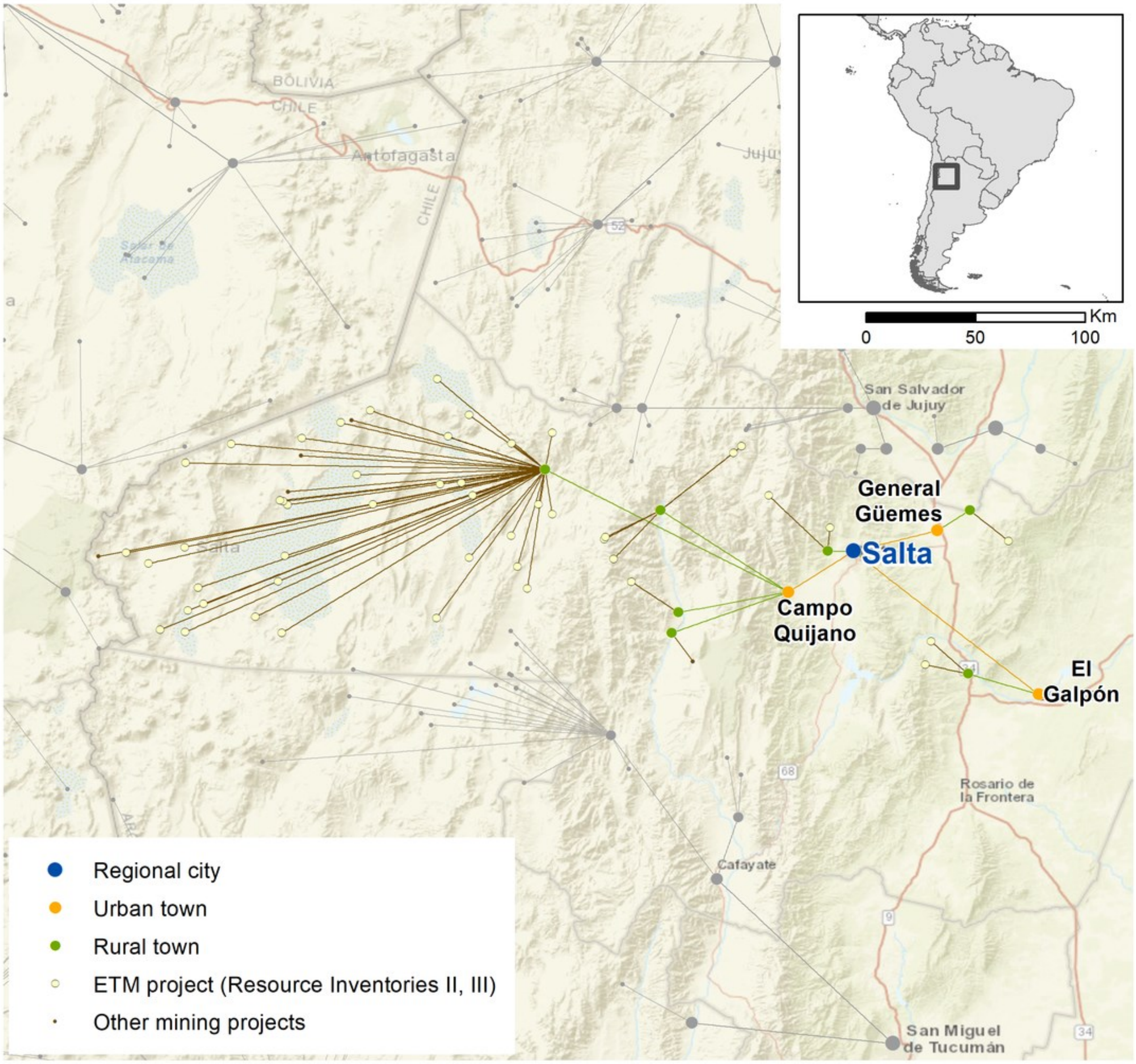

Town system:

- 1 regional city = Salta
- 3 urban towns = El Galpón, General Güemes, Campo Quijano
- 7 rural towns
- 68,500 min. estimated population

Resource Inventories:

- A total of 53 mining projects
- 0 coal projects in Resource Inventory I
- 48 ETM projects in Resource Inventory III: pre-operating 47; operating 1; of which 5 ETM projects in Resource Inventory II: pre-operating 4; operating 1
- 5 other commodities: pre-operating 5

SALTA, ARGENTINA

Salta mine-town system is located in the province of Salta, Argentina. The regional city of Salta (population 618,375 in 2010 census[6]) is the capital of the province of Salta (population 1,214,441 in 2010 census[6]). Salta province records a rapid growth in mining investment, exploration and exports. During the three years between 2019 to 2021, mining employment in Salta doubled[7]. In 2020, the mining sector generated 3,302 direct and indirect jobs, with 90% male employees. Since January 2018, the work in mining grew by 47%. With US$ 2,466 million, Salta received the second highest level of mining investment made by foreign companies in the country[8]. The main focus of the investments is ETMs. In Salta province, mining wages are 1.6 - 3.5 times higher than the average wages in the province. The average remuneration of mining employees was $109,373 in October 2021, a figure higher than the average for Argentina ($104,454).

MINE-TOWN SYSTEM SALTA

The Salta mine-town system is built on ETM Resource Inventories II and III. According to our results, ETM phase-in may impact the entire Salta mine-town system. The ETM phase-in from Resource Inventory II may affect the minimal population of 1,000 people directly (across 2 rural towns) and another 60,000 indirectly (across the urban towns of El Galpón and Campo Quijano and the city of Salta). The ETM phase-in across Resource Inventory III may affect the minimal population of 3,500 people directly in all 7 rural towns, and another 65,000 indirectly in the urban towns of El Galpón, General Güemes and Campo Quijano and the city of Salta.

PHASE-IN, PHASE-OUT ASSUMPTIONS AND POTENTIAL IMPACTS

Pressures on the supply of ETMs are predicted to mount as demands to meet net-zero ambitions expand globally. Salta is one of the mine-town systems where these pressures may intensify. In Argentina, each province is considered the owner of the mineral resources located in its jurisdiction. The three key provinces where ETMs are concentrated (Salta, Jujuy and Catamarca) signed an internal treaty, endorsed by the federal government, in which they created a committee to promote mining investments by setting clear and stable rules. This treaty reinforced the fact that mining is a provincial matter excluding interventions by the federal government[9]. However, political changes in the province may impact the regulation imposed to mitigate the social and environmental impacts of mining. Currently, mine planning in Salta province is driven by environmental rather than socio-economic regulations[10]. If this continues, the communities in Salta mine-town system might be drawn into mining-dominated economy, bringing a rapid population growth that puts pressure on current communities, their basic services, infrastructure and environment (as demonstrated in the current situation in the EMalahleni mine-town system in Example 1). On the other hand, new mining industries may bring new job opportunities and social benefits across the Salta mine-town system. Accruing benefits would require government investment in social services and infrastructure to support burgeoning populations, including new towns to service new mining projects. Each new mine will, at some stage, need to close – leading communities living in the Salta mine-town system back towards the socio-economic and environmental consequences of mine closure or, in the worst case, mine abandonment.

3 | MINE-TOWN SYSTEM EXAMPLE

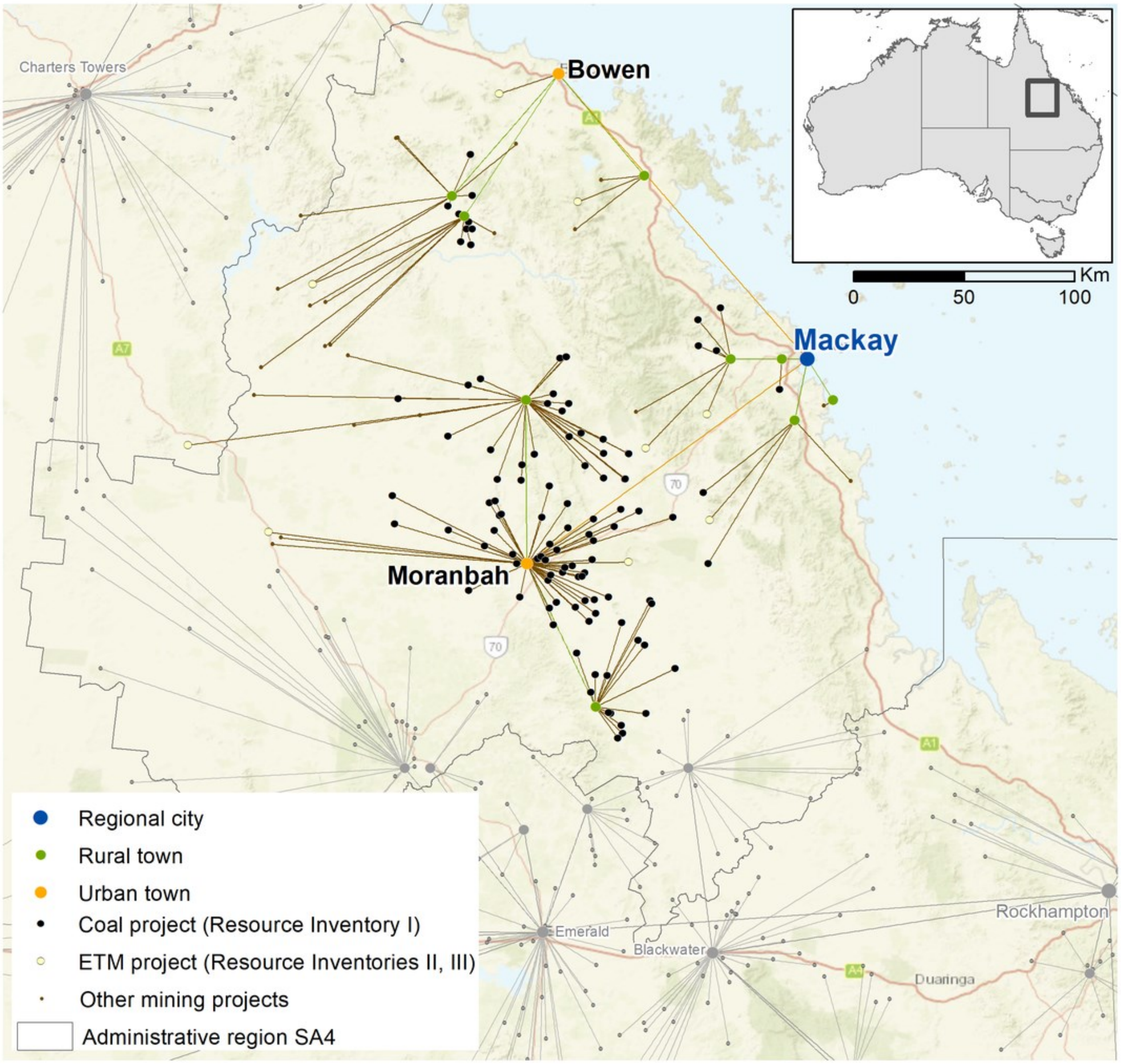

Town system:

- 1 regional city = Mackay
- 2 urban towns = Moranbah, Bowen
- 9 rural towns
- 79,505 min. estimated population

Resource Inventories:

- A total of 138 mining projects
- 106 coal projects in Resource Inventory I: pre-operating 69; operating 32; closed 2; NA 3
- 23 ETM projects in Resource. Inventory III: pre-operating 22; operating 1; of which 6 ETM projects in Resource Inventory II: pre-operating 5; operating 1
- 9 other commodities: pre-operating 8; NA 1

MACKAY, AUSTRALIA

Mackay mine-town system is located in the administrative Mackay-Isaac-Whitsunday region (SA4) in Queensland, Australia. The region is home to 183,269 people, supports 85,165 jobs and has an annual economic output of AU$46.705 billion. It is one of the largest coal and beef producing regions in Australia. Coal mining creates 16.66% of all jobs and 85.4% of all mining jobs in the region. This compares to a national Australian average of 1.7% workers in minerals and energy resources. Income in mining is the highest among all jobs in the region. 55.6% of all mining jobs have a weekly income of more than AU$2,000, compared to agriculture, where this income is only reached by 19% of jobs[11].

MINE-TOWN SYSTEM MACKAY

The Mackay mine-town system is built on all three Resource Inventories. Coal phase-out across Resource Inventory I may impact the minimal population of 7,000 people directly, and another 55,000 indirectly. The impact may spread across 4 rural towns, both urban towns of Moranbah and Bowen and the regional city of Mackay. ETM phase-in from Resource

Inventory II may impact the minimal population of 7,000 people directly, and another 55,000 indirectly, potentially affecting 4 rural towns, urban towns of Moranbah and Bowen and the regional city of Mackay. ETM phase-in from Resource Inventory III may affect the minimal population of 13,000 people directly and another 50,000 indirectly, across 6 rural towns, Moranbah, Bowen and Mackay.

PHASE-IN, PHASE-OUT ASSUMPTIONS AND POTENTIAL IMPACTS

Under the coal phase-out assumption, there may be concentrated job losses in some years due to the scheduled closures of at least 32 currently operating coal mines. These job losses may have knock-on effects for regional economies across the Mackay mine-town system. This may create multiple disruptions to household incomes, local and regional firms heavily tied to coal supply chains, community well-being and social capital, local and regional government capacity, and fiscal solvency. In 9 rural towns across the Mackay mine-town system, the loss of even a dozen jobs may lead to the closure of local elementary schools and lay off teachers. Local health facilities, food markets and other infrastructure may become unviable. With the decline in local services, house prices may fall and people may move to Moranbah, Bowen and Mackay in search of work. Some out-migration may be outside the Mackay mine-town system, as there may not be immediate alternative employment nearby (e.g., Rockhampton and Townsville, the nearest industrial regional cities in Queensland, are approximately 4-5 hours drive from the Mackay mine-town system).

Communities in the settlements inside the Mackay mine-town system may fragment or adjust change their social structure, bringing new social and psychological consequences, including negative effects on mental health and even mortality. Moreover, some types of workers (mainly women, older and less-educated workers) may suffer disproportionately from these disruptions. If these disruptions are unmanaged, they may lead to the loss of assets, skills and social capital across the Mackay-Isaac-Whitsunday region.

Accelerating the shift to the ETM phase-in assumption may cause all 6 ETM mining projects from Resource Inventory II or even all 22 ETM mining projects from Resource Inventory III to become operational. This may bring new job opportunities across the mine-town system, however it is unclear to what extent and how many jobs new ETM mines may offer. In any case, after ETM resource depletion, the phase-in will eventually bring some of the same challenges as the coal phase-out described above.

Supplementary Information 2

Estimated global population in the mine-town systems linked to Inventory II. The table represents the estimated global population in the mine-town systems linked to pre-operational and operational stage energy transition metal projects in Inventory II by commodity (i.e., ETM projects with declared Reserves and Resources). All 17 ETMs listed by Hund et al.[12] are included.

Commodity primary	Population per settlement level	Mine-town systems	
		Directly linked	Indirectly linked
Silver	Rural town	777,000 - 7,770,000	
	Urban town	1,050,210 - 10,500,000	3,420,684 - 34,200,000
	Regional city	>2,650,053	>34,600,692
	Population impacted	>4,477,263	>38,021,376
Copper	Rural town	701,500 - 7,015,000	
	Urban town	950,190 - 9,500,000	3,055,611 - 30,550,000
	Regional city	>2,600,052	>31,900,638
	Population impacted	>4,251,742	>34,956,249
Zinc	Rural town	409,000 - 4,090,000	
	Urban town	610,122 - 6,100,000	21,304,26 - 21,300,000
	Regional city	>1,450,029	>22,700,454
	Population impacted	>2,469,151	>24,830,880
Lead	Rural town	333,000 - 3,330,000	
	Urban town	520,104 - 5,200,000	1,785,357 - 17,850,000
	Regional city	>1,300,026	>19,500,390
	Population impacted	>2,153,130	>21,285,747
Iron Ore	Rural town	230,500 - 2,305,000	
	Urban town	385,077 - 3,850,000	1,115,223 - 11,150,000
	Regional city	>1,300,026	>15,050,301
	Population impacted	>1,915,603	>16,165,524
Nickel	Rural town	159,500 - 1,595,000	
	Urban town	190,038 - 1,900,000	705,141 - 7,050,000
	Regional city	>450,009	>8,800,176
	Population impacted	>799,547	>9,505,317
Molybdenum	Rural town	175,000 - 1,750,000	
	Urban town	300,060 - 3,000,000	985,197 - 9,850,000
	Regional city	>400,008	>12,200,244
	Population impacted	>875,068	>13,185,441
Cobalt	Rural town	137,500 - 1,375,000	
	Urban town	195,039 - 1,950,000	625,125 - 6,250,000
	Regional city	>500,010	>8,000,160
	Population impacted	>832,549	>8,625,285
Vanadium	Rural town	53,000- 530,000	
	Urban town	75,015 - 750,000	320,064 - 3,200,000

	Regional city	>100,002	>3,650,073
	Population impacted	>228,017	>3,970,137
Bauxite	Rural town	45,000 - 450,000	
	Urban town	60,012 - 600,000	230,046 - 2,300,000
	Regional city	>250,005	>3,700,074
	Population impacted	>355,017	>3,930,120
Lithium	Rural town	28,500 - 285,000	
	Urban town	55,011 - 550,000	205,041 - 2,050,000
	Regional city	>50,001	>2,450,049
	Population impacted	>133,512	>2,655,090
Manganese	Rural town	33,500 - 335,000	
	Urban town	80,016 - 800,000	225,045 - 2,250,000
	Regional city	>50,001	>3,000,060
	Population impacted	>163,517	>3,225,105
Titanium	Rural town	35,500 - 35,5000	
	Urban town	35,007 - 350,000	250,050 - 2,500,000
	Regional city	>100,002	>3,000,060
	Population impacted	>170,509	>3,250,110
Graphite	Rural town	30,500 - 305,000	
	Urban town	35,007 - 350,000	170,034 - 1,700,000
	Regional city	0	>2,000,040
	Population impacted	>65,507	>2,170,074
Neodymium	Rural town	8,000 - 80,000	
	Urban town	15,003 - 150,000	50,010 - 500,000
	Regional city	0	>800,016
	Population impacted	>23,003	>850,026
Indium	Rural town	6,000 - 60,000	
	Urban town	0	55,011 - 550,000
	Regional city	>50,001	>550,011
	Population impacted	>56,001	>605,022
Chromium	Rural town	4,500 - 45,000	
	Urban town	5,001 - 50,000	30,006 - 300,000
	Regional city	>50,001	>500,010
	Population impacted	>59,502	>530,016
Aluminium	Rural town	500 - 5,000	
	Urban town	0	5001 - 50,000
	Regional city	0	>50,001
	Population impacted	>500	>55,002

Supplementary Information 3

Mining projects reflecting coal phase-out and ETMs phase-in assumptions relative to mine-town systems. (a, b) Sum of national numbers of coal and ETM projects and directly linked settlements affected by the phase-in and phase-out assumptions. The scatterplots show countries with 20 or more settlements directly connected to coal phase-out and countries with 100 or more settlements directly connected to ETM phase-in assumption.

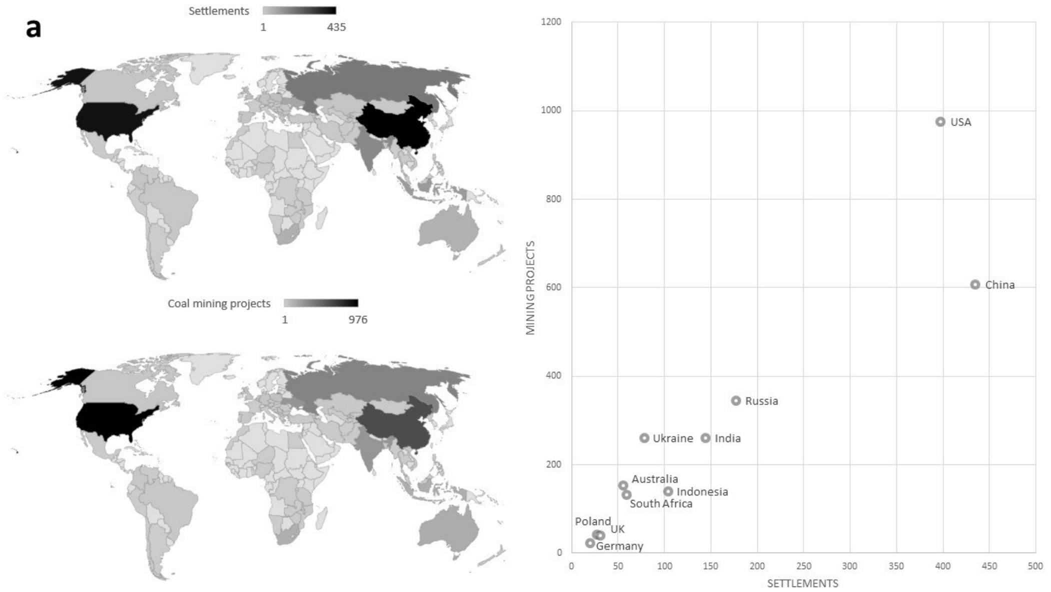

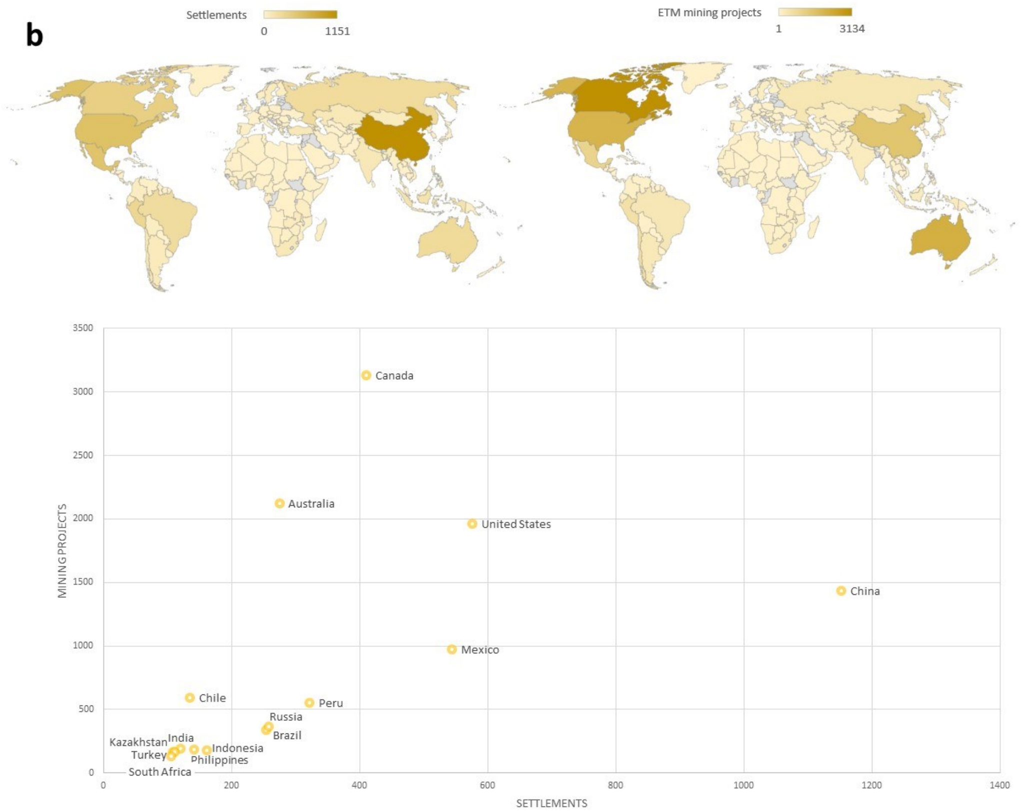

Supplementary Information 4

Global heat maps of the mine-town systems that may be affected by the coal phase-out (a) and ETMs phase-in assumptions (b). The heat maps display the relative density of mine-town systems, ranging from sparse density of points to high density of points. The density is calculated using the Kernel Density method.

a

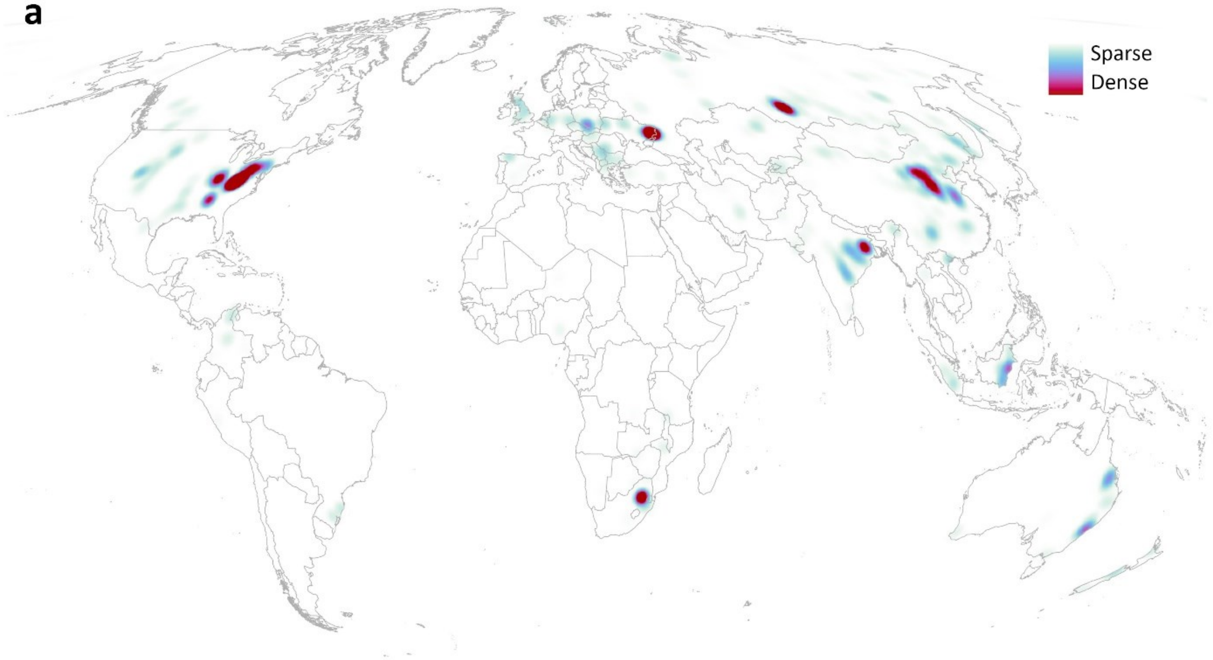

b

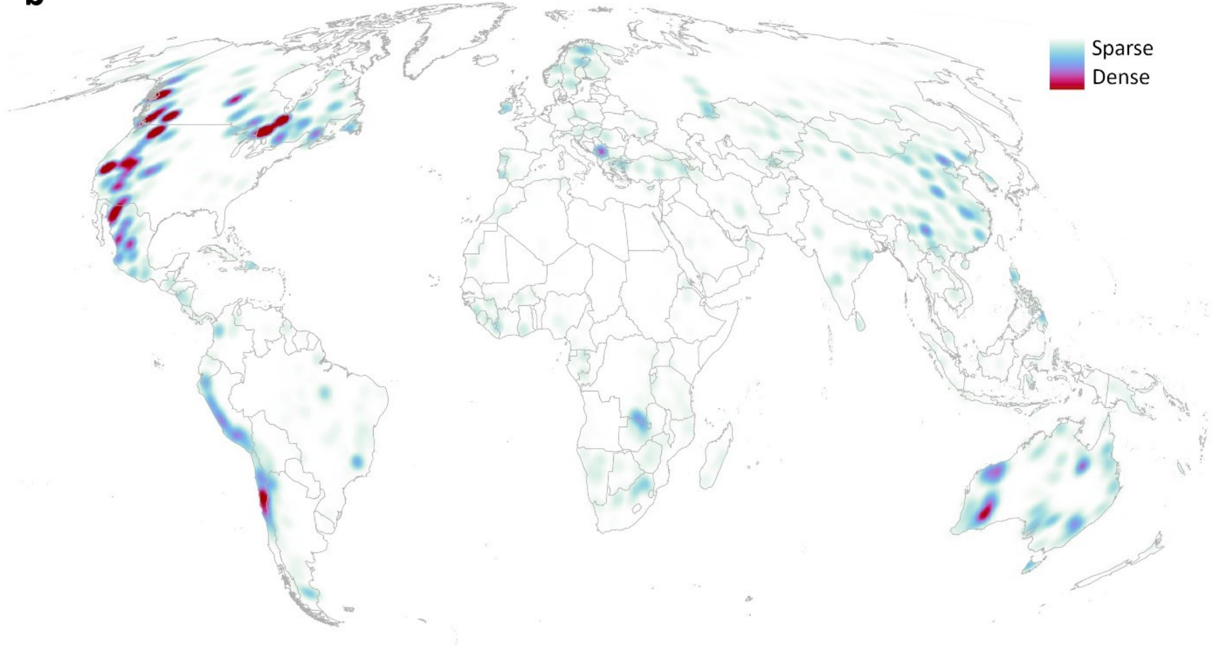

Supplementary references

1. Global Insight. *Regional eXplorer database*. Centurion: HIS Global Insight (2013).
2. Laisani, J., & Jegede, A. O. Impacts of coal mining in Witbank, Mpumalanga province of South Africa: An eco-legal perspective. *Journal of Reviews on Global Economics* **8**, 1586-1597 (2019).
3. StatsSA. *Census 2011*. Pretoria: StatsSA (2012).
4. SACN. Emalahleni: Secondary Cities. *SACN Report under SACN Programme* (2014). https://www.sacities.net/wp-content/uploads/2019/12/Emalahleni-final-report-author-tc.pdf
5. Luthango, S. Extraterritorial Obligations in the Governance Gap. Rosa Luxemburg Stiftung. https://www.rosalux.de/fileadmin/rls_uploads/pdfs/Policy_Paper/Luthango_Policy_Paper_South_Africa_Mine_Closures_1-2022.pdf (2022).
6. Indec. *2010 National Population, Household and Dwelling Census*. https://www.indec.gob.ar/indec/web/Nivel4-Tema-2-41-135 (2022).
7. Government of Salta. En *Salta se incrementó la tasa de empleo minero en más de un 90%* [In Salta, the mining employment rate increased by more than 90%]. https://www.salta.gob.ar/prensa/noticias/en-salta-se-incremento-la-tasa-de-empleo-minero-en-mas-de-un-90-76005 (2021).
8. Government of Salta. Minería en Salta: crecimiento, sustentabilidad y trabajo para los salteños [Mining in Salta: growth, sustainability and work for Salta residents]. https://www.salta.gob.ar/prensa/noticias/mineria-en-salta-crecimiento-sustentabilidad-y-trabajo-para-los-saltenios-80478 (2022a).
9. Dentons. *The Interprovincial Treaty Concerning the Regional Development of the Lithium Sector*. https://www.dentons.com/en/insights/articles/2021/december/9/the-interprovincial-treaty-concerning (2021).
10. Government of Salta. *Environmental and Social Impact Assessment*. https://produccionsalta.gob.ar/producto/evaluacion-de-impacto-ambiental-y-social/ (2022b).
11. ABS. *Table builder census data*. Australian Bureau of Statistics. https://www.abs.gov.au/websitedbs/censushome.nsf/home/tablebuilder (2019).
12. Hund, K., La Porta, D., Fabregas, T. P., Laing, T., & Drexhage, J. *Minerals for climate action: the mineral intensity of the clean energy transition*. World Bank (2020).
