# Supplementary material for: Decarbonization, population disruption and resource inventories in the global energy transition
Source: Nat Commun. 2022 Dec 15;13:7674. doi: 10.1038/s41467-022-35391-2 (PMC9755273; doi:10.1038/s41467-022-35391-2)
Supplement: Supplementary file 3 — Reporting Summary [file 41467_2022_35391_MOESM3_ESM.pdf]

nature portfolio

Corresponding author(s): Kamila Svobodova

Last updated by author(s): Nov 8, 2022

Reporting Summary

Nature Portfolio wishes to improve the reproducibility of the work that we publish. This form provides structure for consistency and transparency in reporting. For further information on Nature Portfolio policies, see our Editorial Policies and the Editorial Policy Checklist.

Statistics

For all statistical analyses, confirm that the following items are present in the figure legend, table legend, main text, or Methods section.

n/a	Confirmed	
☒	☐	The exact sample size (*n*) for each experimental group/condition, given as a discrete number and unit of measurement
☒	☐	A statement on whether measurements were taken from distinct samples or whether the same sample was measured repeatedly
☒	☐	The statistical test(s) used AND whether they are one- or two-sided<br>*Only common tests should be described solely by name; describe more complex techniques in the Methods section.*
☒	☐	A description of all covariates tested
☒	☐	A description of any assumptions or corrections, such as tests of normality and adjustment for multiple comparisons
☒	☐	A full description of the statistical parameters including central tendency (e.g. means) or other basic estimates (e.g. regression coefficient) AND variation (e.g. standard deviation) or associated estimates of uncertainty (e.g. confidence intervals)
☒	☐	For null hypothesis testing, the test statistic (e.g. *F*, *t*, *r*) with confidence intervals, effect sizes, degrees of freedom and *P* value noted<br>*Give P values as exact values whenever suitable.*
☒	☐	For Bayesian analysis, information on the choice of priors and Markov chain Monte Carlo settings
☒	☐	For hierarchical and complex designs, identification of the appropriate level for tests and full reporting of outcomes
☒	☐	Estimates of effect sizes (e.g. Cohen's *d*, Pearson's *r*), indicating how they were calculated

Our web collection on statistics for biologists contains articles on many of the points above.

Software and code

Policy information about availability of computer code

Data collection	MS Excel software was used for collection of mining project data.
Data analysis	All spatial data analyses were conducted using ArcGIS (version 10.7.1).

For manuscripts utilizing custom algorithms or software that are central to the research but not yet described in published literature, software must be made available to editors and reviewers. We strongly encourage code deposition in a community repository (e.g. GitHub). See the Nature Portfolio guidelines for submitting code & software for further information.

Data

Policy information about availability of data

All manuscripts must include a data availability statement. This statement should provide the following information, where applicable:

- Accession codes, unique identifiers, or web links for publicly available datasets
- A description of any restrictions on data availability
- For clinical datasets or third party data, please ensure that the statement adheres to our policy

The data generated in this study are provided in the Source Data file. The dataset sourced from S&P Global Market Intelligence database, that support the findings of this study can be obtained from the corresponding author upon reasonable request. Source links of other publicly-available datasets used for this study (GHS-SMOD, GRUMP and URCA) are provided in References.

Human research participants

Policy information about studies involving human research participants and Sex and Gender in Research.

Reporting on sex and gender	not relevant
Population characteristics	not relevant
Recruitment	not relevant
Ethics oversight	not relevant

Note that full information on the approval of the study protocol must also be provided in the manuscript.

Field-specific reporting

Please select the one below that is the best fit for your research. If you are not sure, read the appropriate sections before making your selection.

☐ Life sciences ☐ Behavioural & social sciences ☒ Ecological, evolutionary & environmental sciences

For a reference copy of the document with all sections, see nature.com/documents/nr-reporting-summary-flat.pdf

Ecological, evolutionary & environmental sciences study design

All studies must disclose on these points even when the disclosure is negative.

Study description

The study presents a novel approach to analysing decarbonisation strategies by linking global resource inventories with demographic systems. Our 'mine-town systems' approach establishes an empirical basis for examining the spatial extent of the transition and demographic effects of changing energy systems. It utilizes the connectivity logic of the Food Web Model. The mine-town systems comprise three hierarchical levels of settlements: rural town, urban town and regional city. By applying a sequential proximity analysis, we modelled mine-town systems analysing socio-economic interactions between settlements and mining projects. The settlement hierarchy determined the point of reference for a given level in the mine-town systems. First, we identified the closest settlement to a mining project. Where rural and urban towns were identified in this interaction, we continued with the second and third sequence of the proximity analysis to indirect interactions until we identified a regional city as the highest hierarchical level of the mine-town system.

Research sample

In mine-town systems, the relationship between mining projects and settlements is a hierarchical network of supply interactions between: (i) mining projects and towns and (ii) towns and cities of different size and importance. These mine-town interactions propagate socio-economic assets and functions throughout the system. To analyse global mine-town systems and their population, the study works with existing datasets on mining projects, settlements and urban-rural catchment areas. We used a global set of 35,891 mining projects sourced from the S&P Global Market Intelligence database, a commercial database that gathers public disclosure data, mainly reported by project owners (S&P 2021; data are current as of January 2021). To generate the hierarchically classified layer of global settlements, we synthesised two data sources: GHS Settlement Model layers (Florczyk et al. 2019) and the Global Rural-Urban Mapping Project: Settlement Points, Revision 01 (CIESIN 2021). The higher a settlement sits in the system hierarchy, the larger its population size and number of socio-economic functions. As socio-economic diversity increases, economic dependence on mining decreases. The dataset of Urban-Rural Catchment Areas (Cattaneo et al. 2021) was used to analyse the remoteness of the rural towns in the mine-town systems.

Sampling strategy

To identify mine-town systems on an international scale, a sample of 35,891 mining projects was sourced from the S&P Global Market Intelligence database (S&P 2021; data are current as of January 2021). We used the most complete project-level records in the S&P, including development stage, commodity and geographical coordinates. Mining projects with missing records on development stage and coordinates were excluded from the analysis, leaving a sample of 35,319 mining projects.
To generate the hierarchically classified layer of global settlements, we synthesised two data sources: GHS Settlement Model layers (Florczyk et al. 2019) and the Global Rural-Urban Mapping Project: Settlement Points, Revision 01 (CIESIN 2021). To reduce the potential for error in geo-locating settlements, we used a 10 km square grid, a distance chosen to better represent the typical physical extent of settlements. Using the 10 km settlement grid cells also greatly improved the speed and control of the computational analysis. Each grid cell containing a settlement was classified according to the hierarchical level of that settlement, with priority assigned to the higher-level settlement. This procedure generated 213,489 cells classified as rural towns, 24,421 cells classified as urban towns, and 9,011 classified as regional cities.
The sequential proximity analysis that determined the mine-town systems was limited to national borders. For each direct socio-economic interaction, we set a threshold of 200 km travel distance as a maximal commuting distance from a settlement to a mining project. Guided by previous studies, we assumed that a maximum of 200 km straight line distance is equal to approximately 2-4 hours of travel time necessary for workers to reach mining projects regularly using ground transportation (depending on local conditions). Our aim was to capture functioning mine-town systems with regular socio-economic flows between towns and project locations. From the sample of 35,319 mining projects, 1,000 projects were located farther than 200 km from a settlement, leaving a final sample of 34,319 projects being part of the mine-town systems.

Data collection

Mining project data was extracted from the S&P Global Market Intelligence directly to an MS Excel file using the S&P database

Data collection	screener and MS Excel add-in. Datasets on global settlements and urban-rural catchment areas were downloaded directly from their online repositories. Data was collected by the corresponding author.
Timing and spatial scale	All data were collected at the beginning of the research project in January 2021 and extracted once from their online repositories. The most recent versions for all datasets were downloaded individually in January 2021. All datasets are global datasets.
Data exclusions	Mining projects with missing records on development stage and coordinates in the S&P database were excluded from the analysis. From this sample, mining projects located farther than 200 km travel distance (200 km straight line distance) from the closest settlement were excluded from the sequential proximity analysis. 200 km straight line distance is equal to approximately 2-4 hours of travel time necessary for workers to reach mining projects regularly using ground transportation (depending on local conditions). The criterion of maximal travel distance excluded fly-in-fly-out commuting schemes. The sequential proximity analysis excluded international connections to avoid cross-national linkages inside the mine-town systems.
Reproducibility	Mine-town systems modelling was performed independently by the first and the fourth authors, reaching the same results. Each author performed it once. The Source Data file contains all data necessary to repeat the experiment, following the same methodological steps and using the same software detailed in the methods section. The study does not generate new data.
Randomization	Randomization is not relevant to the study. The study provides a novel 'mine-town systems' approach by linking existing global resource inventories with existing demographic systems. The allocation of the mining projects and settlements is not random as the data are based on real-world conditions.
Blinding	Blinding is not applicable to the study. This study does not involve any participant and does not rely on possible differential treatment or assessments of outcomes.

Did the study involve field work? ☐ Yes ☒ No

Reporting for specific materials, systems and methods

We require information from authors about some types of materials, experimental systems and methods used in many studies. Here, indicate whether each material, system or method listed is relevant to your study. If you are not sure if a list item applies to your research, read the appropriate section before selecting a response.

Materials & experimental systems

n/a	Involved in the study
☒	☐ Antibodies
☒	☐ Eukaryotic cell lines
☒	☐ Palaeontology and archaeology
☒	☐ Animals and other organisms
☒	☐ Clinical data
☒	☐ Dual use research of concern

Methods

n/a	Involved in the study
☒	☐ ChIP-seq
☒	☐ Flow cytometry
☒	☐ MRI-based neuroimaging
